# Understanding the Influence of API Conformations on Amorphous Dispersion Formation Potential Predictions using the *R*3*m* Molecular Descriptor

**DOI:** 10.1021/acs.molpharmaceut.3c00909

**Published:** 2024-01-05

**Authors:** Kevin DeBoyace, Mustafa Bookwala, Deliang Zhou, Ira S. Buckner, Peter L.D. Wildfong

**Affiliations:** † School of Pharmacy and Graduate School of Pharmaceutical Sciences, Duquesne University, 600 Forbes Ave, Pittsburgh, Pennsylvania 15282, United States; ‡ Drug Product Development, Research and Development, AbbVie, 1 North Waukegan Road, North Chicago, Illinois 60064, United States; § Pfizer Worldwide R&D, Eastern Point Road, Groton, Connecticut 06340, United States; ∥ Small Molecules Drug Product Development, BeiGene USA, Inc., 55 Cambridge Parkway, Cambridge, Massachusetts 02142, United States

**Keywords:** amorphous solid dispersions, molecular descriptors, QSPR, molecular dynamics, molecular conformations

## Abstract

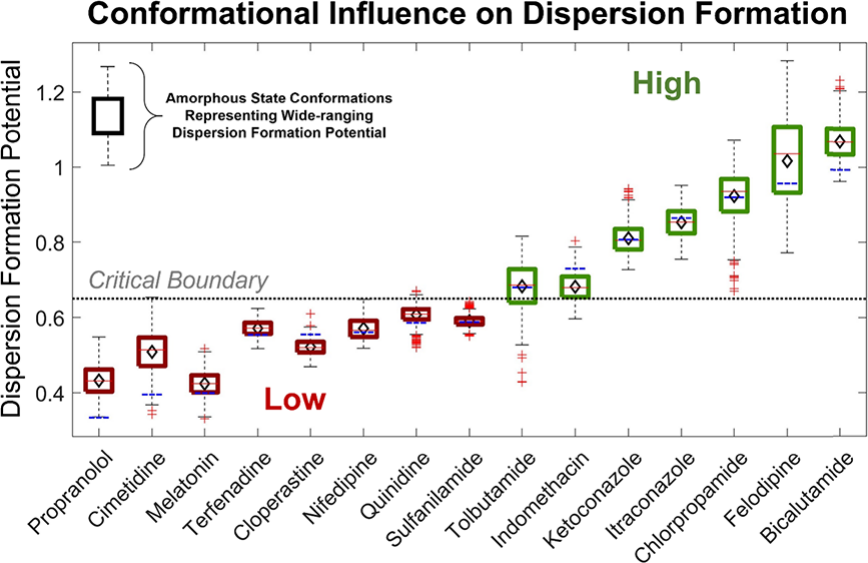

The *R*3*m* molecular descriptor (R-GETAWAY third-order autocorrelation index weighted by the atomic mass) has previously been shown to encode molecular attributes that appear to be physically and chemically relevant to grouping diverse active pharmaceutical ingredients (API) according to their potential to form persistent amorphous solid dispersions (ASDs) with polyvinylpyrrolidone–vinyl acetate copolymer (PVPVA). The initial *R*3*m* dispersibility model was built by using a single three-dimensional (3D) conformation for each drug molecule. Since molecules in the amorphous state will adopt a distribution of conformations, molecular dynamics simulations were performed to sample conformations that are probable in the amorphous form, which resulted in a distribution of *R*3*m* values for each API. Although different conformations displayed *R*3*m* values that differed by as much as 0.4, the median of each *R*3*m* distribution and the value predicted from the single 3D conformation were very similar for most structures studied. The variability in *R*3*m* resulting from the distribution of conformations was incorporated into a logistic regression model for the prediction of ASD formation in PVPVA, which resulted in a refinement of the classification boundary relative to the model that only incorporated a single conformation of each API.

## 1 Introduction

Amorphous solid dispersions (ASDs), which are molecular mixtures of an active pharmaceutical ingredient (API) in a carrier polymer, represent a key formulation development strategy reflected by their employment in several marketed products.^1−4^ The amorphous form of a drug often improves its apparent aqueous solubility relative to the thermodynamically stable crystalline form,^5^ while achieving a single-phase mixture with a polymer adds the potential benefits of improved storage stability relative to the amorphous API and prolonged supersaturation during dissolution.^6^ Although a number of methods have been proposed for the prediction of ASD formation and stability,^7^*a priori* selection of the appropriate API–polymer mixture has proven difficult and the result has been that the majority of ASD formulations have been developed using a trial-and-error approach.

**Figure 1:**
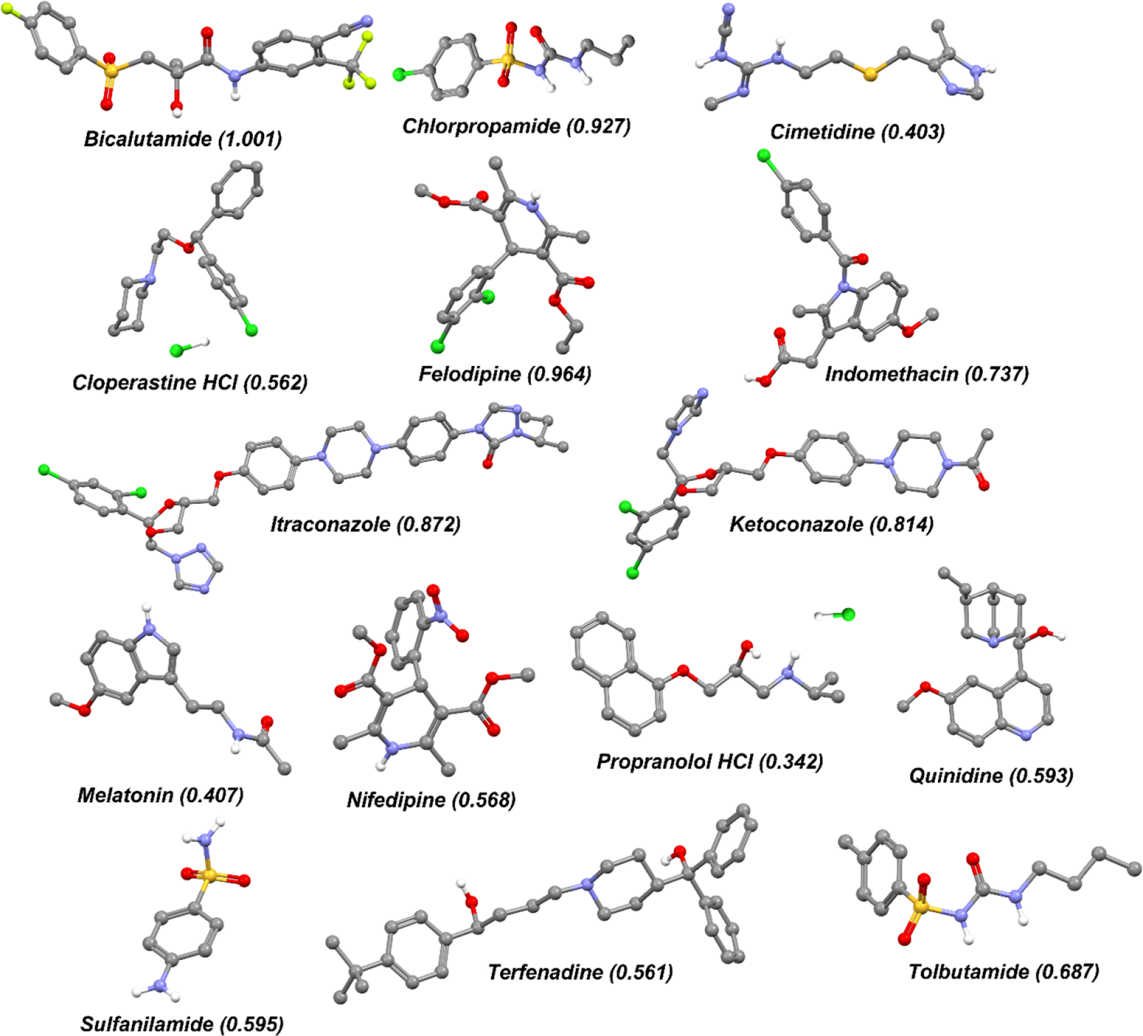
Molecular structures of the 15 API in the compound library generated using the CORINA algorithm. *R*3*m* values based on this conformation are given parenthetically. Hydrogen atoms have been excluded for the sake of clarity, except in the case of hydrogen bond donors.

With the goal of improving over empirical formulation, and thereby reducing development costs and time to market, our research group has pursued molecular descriptors as a materials-sparing tool for the prediction of ASD formation. A single molecular descriptor known as the R-GETAWAY *third-order autocorrelation index weighted by the atomic mass* (*R*3*m*) was previously used to group an 18-molecule library of structurally diverse API according to their ability to form an ASD with polyvinylpyrrolidone–vinyl acetate copolymer (PVPVA), prepared using various methods.^7−9^ When applied to the subset of 15 API that could be prepared using the melt-quench method (Figure 1), it was found that molecules having R3*m* >0.65 all formed persistent ASDs in PVPVA, at both 15 and 75% w/w drug loading, as confirmed using a suite of analytical characterization techniques.^7,8^

*R*3*m* encodes information about the size and shape of an API molecule, its topological connectivity, and the number and relative positions of its atoms in 3D space (with increasing significance for molecules that contain heavier peripheral atoms). The relationship between the physicochemical meaning of *R*3*m* and how it groups molecules according to their ability to form dispersions with PVPVA was thoroughly investigated in a recent publication.^9^ In our previous work, each *R*3*m* value was calculated based on a single molecular conformation, either taken from the reported crystal structure^8^ or generated using the COoRdINAtes (CORINA) algorithm^7^ for each library API. In the present work, it is proposed that, in the amorphous state, each API is likely to adopt multiple conformations, resulting in a distribution of *R*3*m* values, as opposed to one absolute value for each molecule. It was observed that the respective values of *R*3*m* were larger when heavy atoms, capable of forming specific noncovalent interactions with the PVPVA, were peripherally located.^9^ This suggested that conformations in which the 3D coordinates of these key atoms were shifted further from the geometric centers of the API molecules would have correspondingly higher *R*3*m* values. Likewise, conformations where these heavier atoms were positioned closer to the molecular geometric center would have lower *R*3*m* values.

To evaluate the potential range of *R*3*m* that an API might have, molecular dynamics (MD) was used to explore the likely conformations that the library API might adopt in amorphous systems. In this paper, it was hypothesized that the distribution of *R*3*m* values captured by MD-simulated API conformations will be better predictors of dispersibility than a single conformation dictated by either that adopted in a crystal structure or gaseous state minimizations. Although a distribution of *R*3*m* for each molecule was expected to refine the classification boundary relative to its initial value,^7−10^ this approach was expected to result in better estimations of the probability of dispersion formation in PVPVA. To address this hypothesis, the overall impact of 3D conformation on the value of *R*3*m* was investigated. MD simulations were used to obtain a reasonable distribution of conformations expected for the amorphous forms for each library API molecule. Distributions of *R*3*m* were evaluated to see if they contained the original value (calculated from the conformation in the crystal structure). Finally, the model for the prediction of ASD formation was updated to include the distribution in 3D conformations.

## 2 Experimental Section

### 2.1 Preparation of Co-Solidified Mixtures

The preparation of co-solidified mixtures by melt-quenching has been previously described.^7^ Briefly, the polymer and API were mixed at a specific weight ratio and transferred to a crucible, where the mixture was then heated to 10 °C above the API melting point, where it was maintained isothermally for ∼30 min with intermittent stirring. The molten mass was then quenched by immersion of the crucible in ice water under a dry N_2_ purge to minimize exposure to water condensation and plasticization. The resulting solids were stored overnight in a P_2_O_5_ desiccator and characterized the next day by using powder X-ray diffraction (PXRD), differential scanning calorimetry (DSC), and hot-stage polarized light microscopy. Additional details concerning the categorization of dispersibility can be found in the original reference.^7^

### 2.2 Crystal Structure Data

To explore conformational diversity due to different chemical structures beyond the original library shown in Figure 1, 80 individual molecules were identified, consisting of primarily biopharmaceutical classification system (BCS) Class II API. Since the application of this work is highly relevant to ASDs, molecules having poor aqueous solubility were considered as the primary focus to the applicability of the work herein. Crystal structure files were obtained from the Cambridge Crystallographic Data Centre (CCDC)^11^ in order to compare predicted 3D structures to experimental (crystal) data. A total of 130 CCDC files were used, composed of 33 molecules with multiple polymorphic forms and 47 molecules with a single reported crystal structure. The CCDC reference codes for these files can be found in the Supporting Information Table S1. For cases in which multiple crystal structure files existed for a single polymorph, the file with the lowest reported R-factor was selected.

### 2.3 Molecular Dynamics

**Figure 2:**
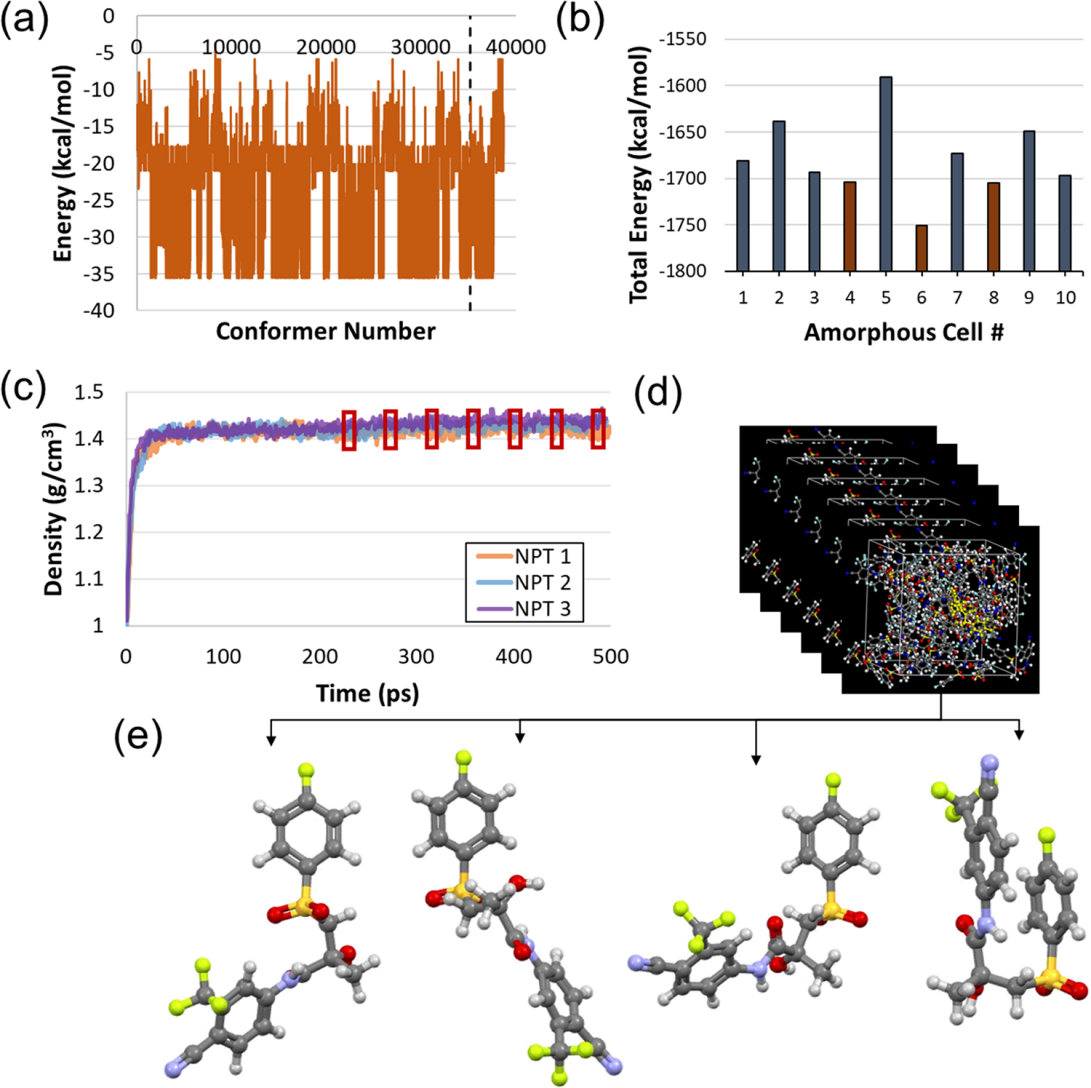
Schematic illustrating the MD simulation for bicalutamide. (a) The *Conformer* tool was used to identify the lowest energy conformation (black dashed line). (b) This conformation allowed construction of 10 unique amorphous cells, where the three having the lowest energy (indicated in orange) were selected for MD simulations. (c) MD simulations using the NPT ensemble allowed selection of multiple frames after equilibration (indicated by the red boxes). (d) Coordinates from the selected frames were extracted using MATLAB allowing calculation of the *R*3*m* values for each individual molecule.

MD simulations were performed *in silico* using Materials Studio 7.0 (Biovia, San Diego, California) to sample multiple 3D structural conformations in the amorphous form. The COMPASS II force field was applied for all of the simulations, since it has been parametrized for drug-like molecules, includes functional groups of interest, and has been optimized using experimental data.^12^ The MD process is illustrated in Figure 2. First, the API structure was drawn and its geometry was optimized using the *Forcite* module. The *Conformers* module was then applied using the systematic grid scan method to identify the conformation of the lowest total energy in a vacuum (Figure 2a). This was performed to ensure that the initial structure for MD simulations was not sterically trapped in an unlikely conformation. Next, 40 molecules derived in the previously identified lowest energy conformation of the API were placed into a virtual cubic “box” having a density of 1 g/cm^3^ using the *Amorphous Cell* module. The construction of the amorphous cell was replicated to generate a total of 10 unique cells, and the three cells having the lowest total energy following geometry optimization were selected for subsequent MD simulations (Figure 2b). Finally, the *Forcite* module was used to complete MD simulations for a total of 500 ps by using the NPT ensemble to permit the cell volume to fluctuate (Figure 2c). The virtual temperature was held at 298 K using the Nosé–Hoover thermostat, and the virtual pressure was held at 1 atm using the Berendsen barostat. The amorphous density for each API was determined by taking the average of the density over the final 300 ps of the simulation, which was previously shown to result in accurate estimations of this parameter.^13^ Seven equally spaced frames were selected (Figure 2d) throughout the final 300 ps of the simulations (illustrated by the red boxes in Figure 2c). Individual molecular conformations were then extracted from these frames, and an *R*3*m* value was calculated for each conformation (Figure 2e).

MATLAB was used to extract the 3D molecule conformations from the Materials Studio MD simulations. Seven frames per simulation were saved as as.xsd files, where relevant data, such as the atom types, atom coordinates, and connectivity, were obtained using an additional MATLAB code, developed in-house. Coordinates for each molecule were centered, and the *R*3*m* descriptor was calculated for each conformation using another in-house MATLAB function. The MATLAB functions for this process can be obtained via the links provided in the Supporting Information.

## 3 Results and Discussion

*R*3*m* belongs to the geometry, topology, and atom weights assembly (GETAWAY) class of descriptors, whose calculation is described in greater detail elsewhere.^9,14,15^*R*3*m* is calculated using eq 1(15):
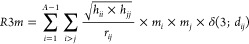
1where *A* is the number of atoms in the molecule, *m* is the atomic mass normalized to carbon, *i* and *j* refer to two separate atoms, *r_jj_* is the Euclidean distance between them, *h_ii_* and *h_jj_* are the leverages, and δ(*k*:*d_ij_*) is a delta-Dirac function, where *k* = 3 restricts the calculation to atoms separated by a topological distance of 3. Calculation of *R*3*m* uses several matrices. The first is the 3 × A molecular matrix, which contains the atomic coordinates. The *A* × *A* symmetric geometry matrix contains the Euclidean distance between each atom in the molecule and is calculated in the same manner as the leverage matrix for linear regression,^14^ where its diagonal contains the leverage values (i.e., *h_ii_*, *h_jj_*) used in eq 1.

In the present work, probable 3D conformations of a molecule in the amorphous state were used to evaluate their impact on the matrices comprising eq 1. Changing the conformation of a molecule results in a change to the values of the molecular matrix for an API and subsequently its geometry matrix. While the Euclidean distance of atoms having a topological distance of *k* = 1 (i.e., atoms connected by a covalent bond) will essentially remain the same, owing to the consistency of these bond lengths, the Euclidean distances of atoms separated by larger topological distances will vary when conformational differences result in altered torsion and bond angles. Likewise, leverage values, which measure the distance of atoms from the geometric center of a molecule, will also change when torsion angles cause significant changes in the location of the geometric center of a molecule. This reinforces that changes in 3D conformation should result in changes to *R*3*m*; however, the extent and significance of these changes depend on the structure. It was, therefore, necessary to first investigate whether conformational changes could result in any significant changes to *R*3*m*, and by extension, have a potential impact on future predictions of ASD formation in PVPVA made by the original *R*3*m* model.^9^ It is also noted that previous investigations of *R*3*m* in the context of dispersions formed via solvent-based cosolidification (e.g., spray-drying) have revealed that residual solvent can affect drug–polymer miscibility and, therefore, is likely to affect the conformations of API in the amorphous state.^7,8,10^ For simplicity, therefore, only binary drug–PVPVA systems prepared via melt-quenching were considered here and extrapolation of the present findings to ASDs prepared using solvent-based methods should be done with caution.

### 3.1 Investigating the Impact of 3D Conformation

Since the 3D coordinate system of an API impacts the magnitude of its *R*3*m*, it was important to consider the source of atomic coordinates. The 3D coordinates generated by pseudoforce field predictions and MD simulations were compared against experimental data to investigate the variability in *R*3*m* caused by variations in 3D coordinates.

#### 3.1.1 CORINA vs Crystal Structure Data

Initially, *R*3*m* was calculated using coordinates from API conformers extracted from reported crystal structures of marketed forms; however, to make the original molecular descriptor model more generally applicable, 1D data from the Simplified Molecular Input Line Entry Specification (SMILES) files were used to predict 3D conformations. The 3D conformations of the 15 library API shown in Figure 1 were predicted using the CORINA algorithm,^16^ which sets bond angles and lengths based on the types of atoms, hybridization states, and bonds. Additionally, atom positions are adjusted to avoid nonbonded atom overlap and a pseudoforce field is applied to minimize the sum of stretching, bending, out-of-plane, and torsional energies.^16^ CORINA has been shown to make reasonable predictions of molecular conformations in crystals,^16^ where a data set composed of 2443 small organic molecules resulted in an average root-mean-square deviation between actual and predicted atomic coordinates of 0.95 Å. The shift from crystal structure conformations to CORINA-predicted conformations was made so that the *R*3*m* model could be applied to new chemical entities without solved crystal structures. This change in approach also provided an initial evaluation of how sensitive *R*3*m* values were to conformational changes, in general.

**Figure 3:**
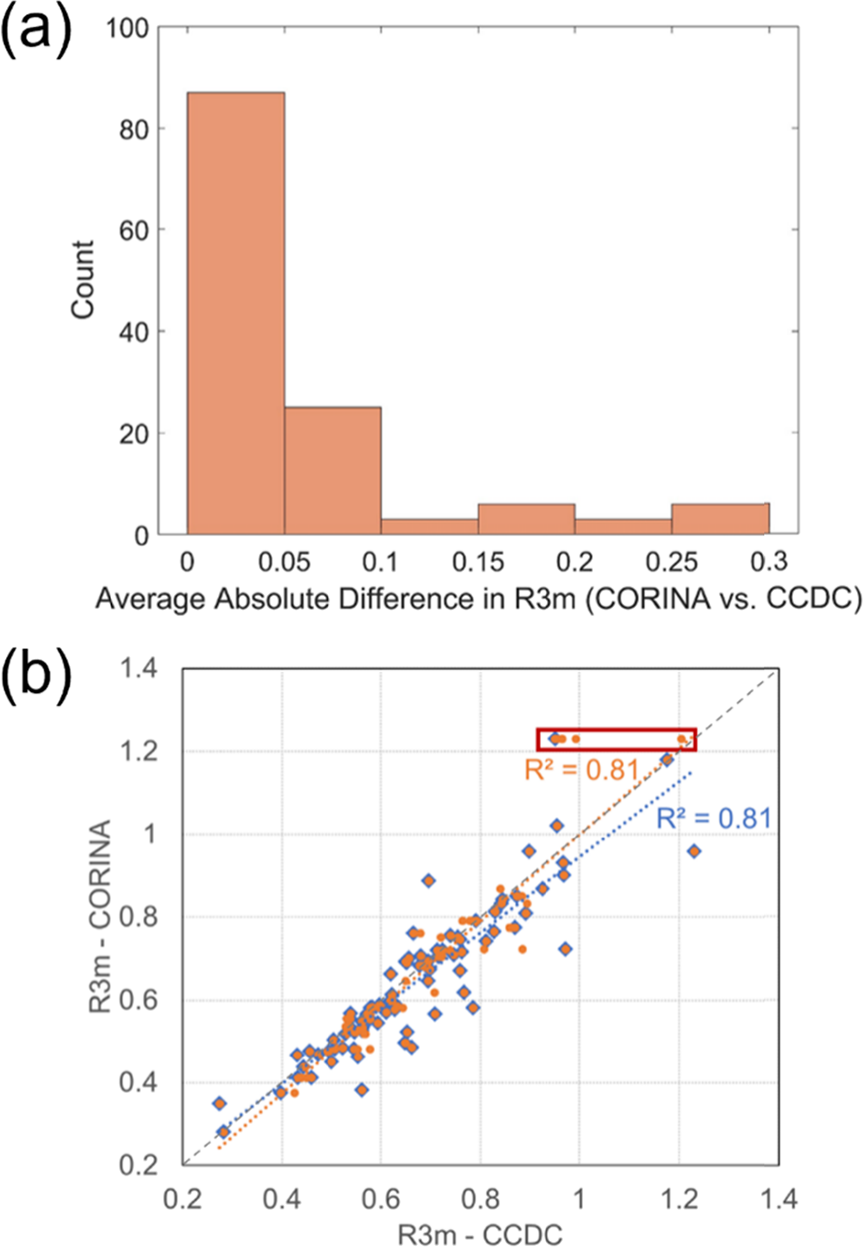
Histograms showing the average absolute difference between *R*3*m* values calculated from CORINA-predicted and CCDC obtained structures. (a) One hundred thirty API, including *R*3*m* from 50 polymorphic structures. Linear relationship between (b) *R*3*m* values for 80 API (blue diamonds) and 50 polymorphs totaling 130 structures (orange circles). The orange regression line reflects the fit for all 130 structures. Points enclosed by the red box specifically highlight the different *R*3*m* values calculated for the polymorphs of aripiprazole. Gray dashes indicate the parity line.

An initial comparison was undertaken using coordinates from both the reported crystallographic structures and from CORINA predictions. To investigate how the value of *R*3*m* varies with conformation for a diverse set of molecular structures, a data set of primarily BCS Class II crystalline structures was assembled (see Table S1 in the Supporting Information for a list of API and their respective CCDC reference codes). Of these 80 API, 33 molecules had reported more than one polymorphic form (ranging from two to eight forms), allowing *R*3*m* to be determined from more than one conformation of these molecules. Inclusion of these data allowed for the assessment of the effect of 3D conformational variability on *R*3*m* within individual API. Histograms reporting the average absolute difference between calculated *R*3*m* values are shown in Figure 3a, which includes a total of 130 molecules (50 polymorphs from 33/80 original structures). A low average absolute difference between *R*3*m* values was observed for most molecules, where the difference in *R*3*m* between CORINA and CCDC 3D coordinates was ≤0.05 for 67% (87/130) of the 130 structures (API and polymorphs), although 4% (5/130) differed by almost 0.3. Figure 3b regresses the 130 structures that included polymorphs and have respective slopes of 0.91 and 1.04. In either case, 81% of the variation in *R*3*m* values calculated from CORINA conformations was explained by variation in *R*3*m* values from CCDC conformations.

**Figure 4:**
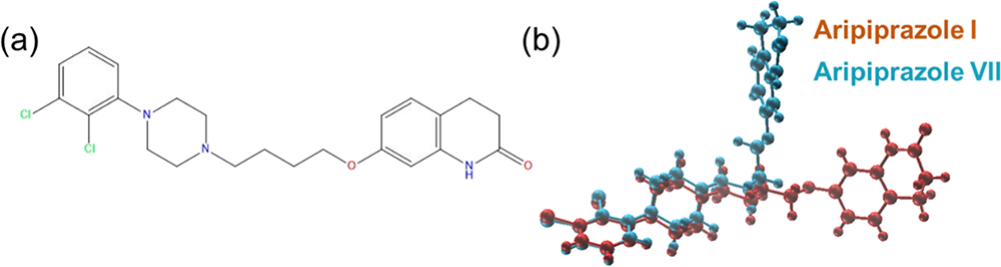
(a) Molecular structure of aripiprazole. (b) CCDC structures for two aripiprazole polymorphs, overlaid on the dichlorophenyl groups. The torsional differences changed the 3D coordinates, resulting in a relatively significant difference in *R*3*m* values: aripiprazole I (CCDC ID: MELFIT01,^17^ orange) and aripiprazole VII (CCDC ID: MELFIT07,^18^ teal).

While the *R*3*m* difference between the CORINA and CCDC conformations was generally small, there were some notable exceptions. One such example was aripiprazole (see red box in Figure 3b), which had the largest observed difference between the calculations of *R*3*m* from CCDC and CORINA conformations (Δ*R*3*m* = 0.28). For comparison, aripiprazole has several polymorphs in the CCDC with different *R*3*m* values. Figure 4a shows the aripiprazole molecular structure, while Figure 4b shows the 3D coordinates from CCDC crystal structures for its polymorphs having the largest difference in their calculated *R*3*m* values. Polymorphs I and VII, respectively, had dihedral angles of 174.7 and 58.34°, representing significant torsional differences between their linear butyl carbon chains. Correspondingly, the different geometric centers for each conformation resulted in changes to the contribution of the matrices for leverage, specific atomic positions, and, ultimately, the Euclidean distances between atoms, which resulted in *R*3*m* = 0.951 (form I) and *R*3*m* = 1.204 (form VII).

Although the average absolute difference in *R*3*m* for all molecules studied was 0.06, the potential for variation in *R*3*m* with conformation was clear: any calculated value of *R*3*m* may vary significantly from the “true” *R*3*m* value. Theoretically, the “true” value would reflect the real 3D conformation of the API in the environment of interest, yet the conformation of molecules in an amorphous system is likely more variable than in vacuum or a crystal. This was especially important for the original *R*3*m* model for ASD formation in PVPVA, which was established by using molecular conformations from API crystal structures. Since dispersion behavior was predicted based on a classification boundary value of *R*3*m* = 0.65, conformational variability of API having an *R*3*m* close to 0.65 could potentially allow the range of calculated *R*3*m* values to fall on either side of this boundary, resulting in different dispersibility predictions. In these cases, it seems that the distribution of possible conformations could be particularly important.

### 3.2 Simulation of Amorphous 3D Conformations Using Molecular Dynamics

Capturing information about the conformations of molecules in the amorphous state is experimentally challenging (if not impossible). The use of MD simulations, therefore, provides an *in silico* means of investigating the effects of probable API conformations on the formation of dispersions. Ideally, since the value of *R*3*m* can be used to predict the formation of ASDs in PVPVA, the calculated values of *R*3*m* should reflect the distribution of conformational possibilities for the API that are most likely in the amorphous form. By determining the breadth of *R*3*m* distributions for each API in our library, it was possible to refine the dispersion prediction model and comment on the likelihood of misclassified dispersion behavior for molecules close to the decision boundary.

Since experimental data for the 3D conformations likely in the amorphous form were not available, MD simulations were applied to predict these structures, as described in Section 2.3. Triplicate 500 ps simulations were run for each API, and individual simulation frames were extracted from seven frames equally spaced throughout the final 300 ps of the simulation (i.e., after 200 ps, where the system appeared to reach equilibrium). A sampling of possible 3D conformations allowed extraction of the 3D coordinates from individual molecules, and *R*3*m* was calculated for each. The distribution of *R*3*m* values for molecules in the amorphous state was then constructed from a total of 840 conformations for each API. To ensure that the output of MD simulations was meaningful, the amorphous density of simulated materials was compared with the experimental amorphous density values (Table 1).^13^

**Table 1:** Predicted and Actual (where available) Amorphous Densities for Library API

**API**	**CORINA *R*3*m***	**experimental amorphous density**(g/cm^3^)	**predicted amorphous density**(g/cm^3^)	**95% crystalline density**	**crystallographic density**(g/cm^3^)	**CCDC refcode**
propranolol	0.342	N/A	1.08 ± 0.004	1.106	1.164	IMITON^19^
cimetidine	0.403	N/A	1.20 ± 0.001	1.246	1.312	CIMETD^20^
melatonin	0.407	N/A	1.16 ± 0.008	1.212	1.276	MELATN01^21^
terfenadine	0.561	N/A	1.04 ± 0.016	1.07	1.13	EWEMIF^22^
cloperastine	0.562	N/A	1.11 ± 0.003	N/A	N/A	N/A
nifedipine	0.568	1.20^a^	1.23 ± 0.004	1.313	1.382	BICCIZ03^23^
quinidine	0.593	1.17 ± 0.009^b^	1.14 ± 0.002	1.172	1.234	BOMDUC^24^
sulfanilamide	0.595	N/A	1.44 ± 0.016	1.438	1.514	SULAMD03^25^
tolbutamide	0.687	N/A	1.21 ± 0.007	1.189	1.252	ZZZPUS18^26^
indomethacin	0.737	1.31^c^	1.29 ± 0.002	1.303	1.372	INDMET^27^
ketoconazole	0.814	1.27 ± 0.006^b^	1.30 ± 0.003	1.330	1.4	KCONAZ^28^
itraconazole	0.872	1.27^d^	1.26 ± 0.015	1.292	1.36	TEHZIP^29^
chlorpropamide	0.927	N/A	1.36 ± 0.003	1.378	1.45	BEDMIG10^30^
felodipine	0.964	1.28^a^	1.26 ± 0.005	1.378	1.451	DONTIJ^31^
bicalutamide	1.001	N/A	1.43 ± 0.009	1.476	1.554	JAYCES^32^

a Marsac et al.^33^

b Bookwala et al.^13^

c Tong and Zografi^34^

d Six et al.^35^

Previously, it was shown that the experimentally determined amorphous densities for 10 small-molecule organic solids were very comparable with MD-simulated amorphous densities, having an average percent error of −0.7%. When compared with estimates of amorphous density using a previous suggestion that takes 95% of the crystallographic density,^33,36,37^ the values differed by an average percent error of +3.7%.^13^ This suggested that the MD simulations captured molecular arrangements in a volume close to that of the real amorphous form. This result helped to indicate that MD-simulated amorphous densities were reasonable approximations for materials that could not be experimentally rendered amorphous. Consequently, simulations of molecular conformations in realistic amorphous “cells” lent realism to their use in this work and all amorphous densities used were confirmed to be always lower than the corresponding crystallographic density obtained from crystal structures where available.

### 3.3 Calculating the Distribution of *R*3*m*

**Figure 5:**
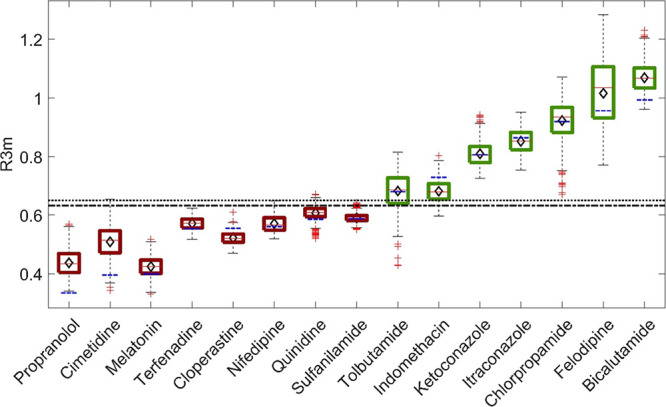
A box-and-whisker plot for *R*3*m* values calculated from MD simulations for each API. Red lines toward the center of each box are median values, while the black diamonds indicate the mean. Each box boundary represents the interquartile range, with whiskers indicating the range of observed values. Outliers are shown using red “+” symbols, and the blue dashed lines indicate *R*3*m* values calculated from SMILES files and the CORINA algorithm. The horizontal black dotted line indicates the *R*3*m* = 0.65 classification boundary published previously,^8,9^ and the horizontal black dot-dashed line indicates the boundary updated using MD simulation results (*R*3*m* = 0.632, see Figure 8). Green boxes indicate API experimentally observed to successfully form ASDs in PVPVA, while those in red indicate compounds that failed to form ASDs.^7−10^

The box-and-whisker plot shown in Figure 5 reports the 840 *R*3*m* values calculated by sampling conformations of 15 API molecules simulated by MD. Median *R*3*m* values determined using MD simulations for each molecule were generally similar to CORINA-predicted values and *R*3*m* calculated from crystal structure conformations used to build the original dispersibility model.^7−9^ The absolute difference between the single *R*3*m* calculated from CORINA structures and the medians of *R*3*m* distributions resulting from MD was taken, resulting in an average absolute difference of 0.03 for the library. The largest of these absolute differences was for cimetidine (0.11), where the variability observed for this molecule specifically resulted from the flexibility of the chain extending from the imidazole group, which caused variations in the Euclidean distances between the intramolecular S and the two nearest N atoms (see Figure 1).

**Figure 6:**
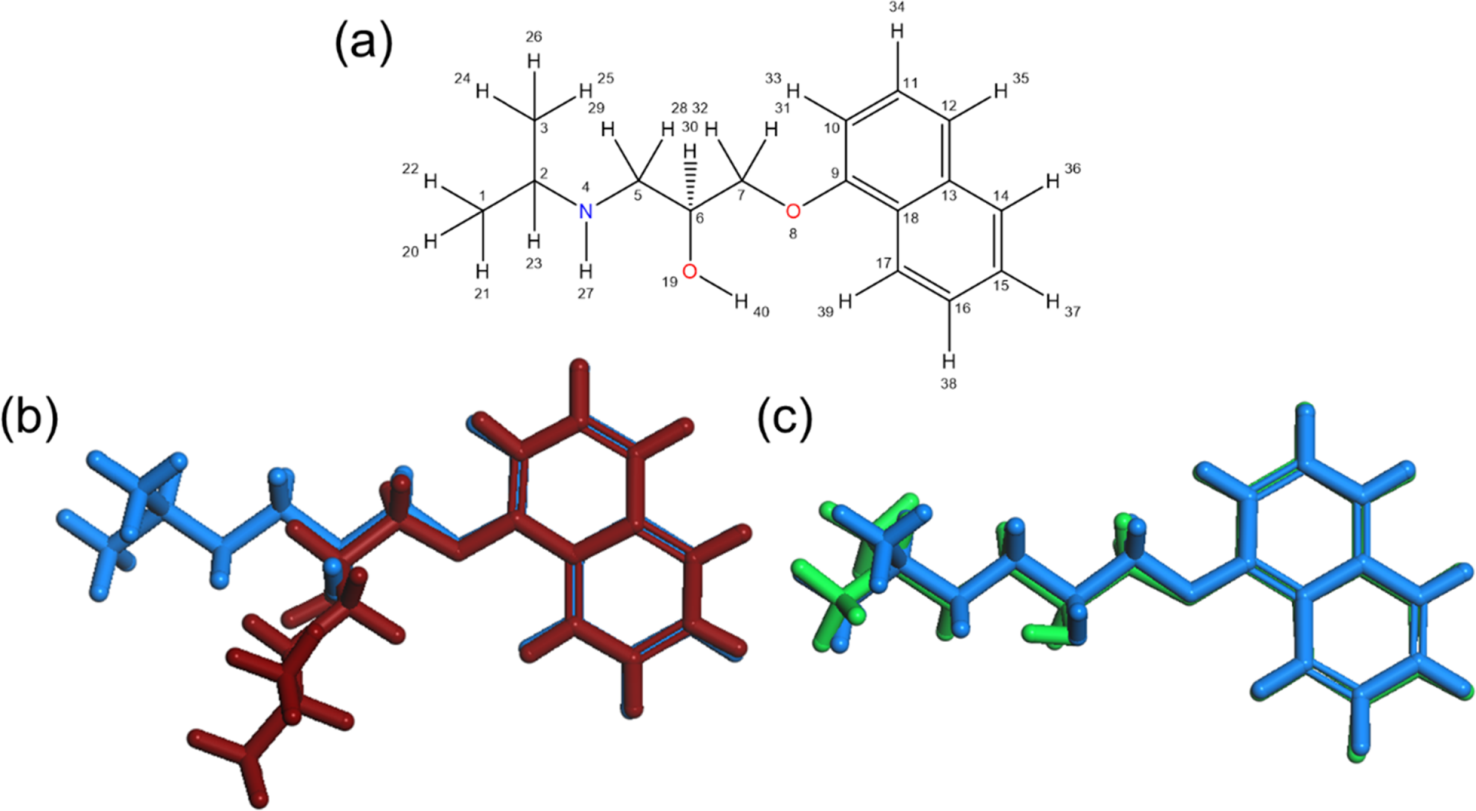
Propranolol conformations and their effect on *R*3*m*. (a) Propranolol structure with atoms labeled. Propranolol molecules in blue correspond to the CORINA calculated conformation ((b) and (c)). (b) The propranolol molecule in red was extracted from MD simulations and has an *R*3*m* value equal to the median (0.4342). (c) The green propranolol molecule shows a conformation from the MD simulation having an *R*3*m* value (0.3406) most similar to the CORINA conformation (0.342) (difference in *R*3*m* = −0.0014).

Values of *R*3*m* from CORINA-predicted conformations all fell within the range of MD simulations with the exception of propranolol (Figure 5). The CORINA algorithm predicted a linear conformation for propranolol, shown by the blue molecule (Figure 6b,c). The red conformation, also shown in Figure 6b, was taken from the MD simulation and had an *R*3*m* value matching the median for the distribution. The green conformation in Figure 6c was also extracted from the MD simulation, based on its similarity to the *R*3*m* value calculated using the CORINA algorithm (difference in *R*3*m* = 0.0014). A linear conformation was less commonly observed in the MD simulations owing to the impact of the surrounding environment. In contrast, rotation about the O8, C7, C6, and C5 dihedral was more common and resulted in an increase in *R*3*m* relative to that calculated for a linear conformation. Numerically, the *R*3*m* value changes due to the resulting shift in the geometric center of the molecule and subsequent change in leverage of each atom.

These results increased the confidence that the *R*3*m* values determined using the conformations predicted from the CORINA algorithm were sufficiently representative of molecules in the amorphous state. The *R*3*m* values applied to build the original dispersibility model all fell within the distributions calculated using conformations obtained from the MD simulations. This indicated that the *R*3*m* values used to build the original model captured relevant and realistic structural information for the API molecules, relative to how they might appear during ASD formation with PVPVA.^9^ Importantly, the MD-determined *R*3*m* values allow dispersibility predictions to shift away from a phenomenological dichotomy and embrace the fact that molecules may adopt conformations that make dispersion in PVPVA more or less probable. As such, the distribution of *R*3*m* for a molecule can help incorporate the relative risk of an incorrect prediction based on probabilities that deviate from 1 or 0. A bar plot that compares the *R*3*m* values calculated from CORINA-predicted structures, conformations extracted from CCDC structures, and MD simulated structures is shown in Figure S1of the Supporting Information.

### 3.4 Updating the *R*3*m* Model

To account for the large number of potential molecule conformations extracted from the present MD simulations, their respective *R*3*m* values were modeled against experimental observations of dispersion formation in PVPVA, using logistic regression, resulting in eq 2.
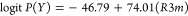
2

**Figure 7:**
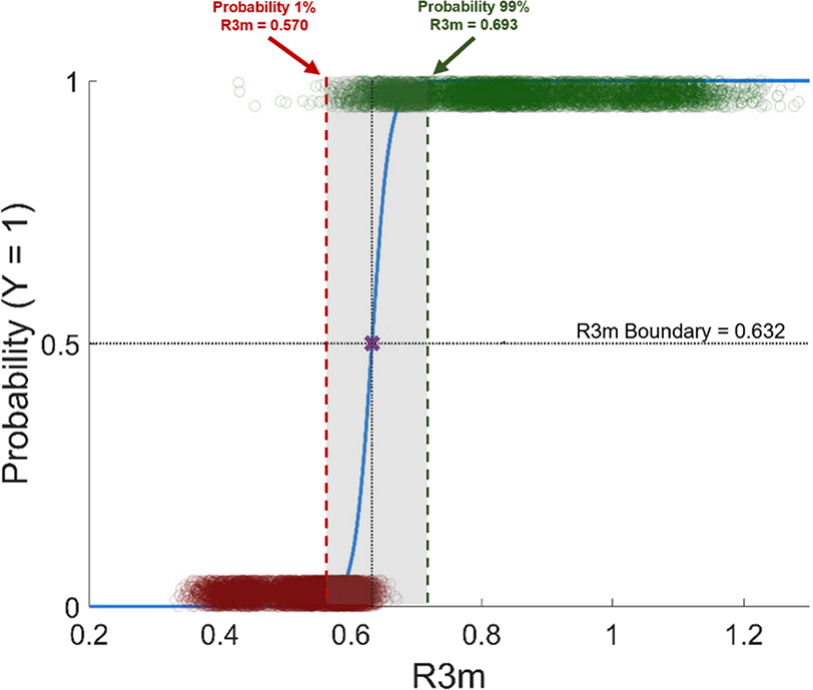
Logistic regression model logit *P*(*Y*) = −46.79 + 74.01(*R*3*m*) (blue line) using the expanded MD data set, resulting in an updated classification boundary value of *R*3*m* = 0.632. This model contains 12,600 data points for *R*3*m* (jittered to more clearly show the amount of data). Red circles correspond to API that failed to form ASDs in PVPVA, while green circles correspond to API that successfully formed ASDs in PVPVA by melt-quenching. The red dashed line indicates that molecules having R3*m* <0.570 have <1% probability to disperse in PVPVA, while the green dashed line indicates that molecules having R3*m* >0.693 have >99% probability to disperse in PVPVA.

All *R*3*m* values associated with API that were observed to disperse in PVPVA were assigned a probability of 1 and all API that did not disperse were assigned probability of 0, and the model remained highly significant having *p* < 0.0001, a pseudo *R*^2^ = 0.91, and χ^2^ = 1.52 × 10^4^. As Figure 7 shows, this leads to some overlap between the *R*3*m* values associated with successful or unsuccessful dispersion formation. Applying logistic regression to these data predicted that structures having an *R*3*m* = 0.632 had a 50% probability of successfully forming a dispersion in PVPVA. Furthermore, according to this MD-based model, *R*3*m* values below 0.570 had a probability <1% and values greater than 0.693 had a probability >99% of forming a homogeneous amorphous dispersion with PVPVA. Expansion of the *R*3*m* model in this way affords some evaluation of the risk associated with attempting to disperse an API in PVPVA, enabling its potential use as a formulation tool. While *a priori* application would be difficult, especially for API having a median R3*m* < 0.632, but some conformers with *R*3*m* above this threshold, it is anticipated that the *R*3*m* model could serve as the first step in a materials-sparing process that also included experimental determination of dispersibility behavior. Take for example the cases of melatonin and quinidine. The values in the distribution of *R*3*m* for melatonin all fall well below the 0.632 categorical boundary (Figure 5). Combined with the probabilities shown in Figure 7, melatonin is very unlikely to ever form a dispersion in PVPVA, consistent with experimental observations under various conditions.^7−9^ A new API having similar data would, therefore, make a poor candidate for ASD formation using this polymer. In contrast, the data for quinidine show that a small portion of its conformer distribution has values of R3*m* > 0.632. A new API having MD-simulated data similar to quinidine might merit experimental confirmation of dispersibility in PVPVA, especially if the therapeutic implications of the drug were promising, and aqueous solubility was its main limitation. Although experimentally, quinidine was never found to disperse in PVPVA,^7−9^ use of a materials-sparing tool such as the *R*3*m* model could additionally point toward the inclusion of a ternary dispersion-enabling component, should the molecular descriptor suggest that there are conformations that make dispersion in the desired polymer feasible.

In the original model, all API having R3*m* <0.6 failed to form ASDs while those having R3*m* >0.68 successfully formed ASDs in PVPVA.^38^ When each molecule was constrained to a single conformation, the completely separated data made logistic regression unnecessary and inappropriate.^39^ Instead, the classification boundary (*R*3*m* = 0.65) that grouped molecules according to dispersibility in this copolymer was defined as the midpoint between the values at the edge of each phenomenological category. As shown, the new *R*3*m* model boundary value shifted to 0.632 from the previously reported value of 0.65, which is illustrated by the horizontal dotted (*R*3*m* = 0.65) and dot–dash (*R*3*m* = 0.632) lines in Figure 5. When classifications of the dispersibility of the 15 library molecules in PVPVA were made using the updated model boundary using either the CORINA-predicted *R*3*m* or the median from the *R*3*m* distributions, they remained unchanged relative to the original model.

In the present case where *R*3*m* adopts a distribution of values for each API, there is uncertainty in the true boundary because of the gap between *R*3*m* values of 0.595 (sulfanilamide) and 0.687 (tolbutamide). Although the updated boundary (*R*3*m* = 0.632) is based on a larger data set that includes the distribution of 12,600 3D conformations, the number of unique API remains relatively small (*n* = 15). Incorporation of additional molecules (and their probable conformers) whose *R*3*m* distributions are close to 0.632 is expected to improve the uncertainty around the value of the categorical boundary; nonetheless, the classification of dispersion behaviors for each API using the original model remains unchanged. The similarity between the models further supports the conclusion that the *R*3*m* descriptor is able to capture relevant 3D information about the API. Incorporation of these additional data into the model resulted in improved confidence in the model boundary by ensuring that the model incorporates conformational flexibility. As a result, future predictions will likely improve, particularly for API with *R*3*m* values near the model boundary.

### 3.5 Marketed API Misclassification by the *R*3*m* Model: Ritonavir and Lopinavir

It was previously identified that ritonavir and lopinavir had CORINA-based *R*3*m* values <0.632, respectively, at *R*3*m* = 0.611 and *R*3*m* = 0.588. These values indicate that ritonavir and lopinavir group with other APIs whose molecular attributes make the formation of persistent dispersions with PVPVA less likely.^9^ Since both ritonavir and lopinavir are marketed ASD products, it has already been demonstrated that these molecules have an ability to persist as an ASD with this copolymer^9^; however, it is important to note that these products also contain surfactants, albeit at relatively low concentrations. Additionally, the marketed ASD drug products of ritonavir and lopinavir have very low drug loadings.^40^ Although the applicability of the *R*3*m* model to dispersions having more than two components and drug loadings less than 15% have not been studied, MD simulations of these molecules were also performed to (1) see what percentage (if any) of likely conformations for either molecule in the amorphous phase resulted in R3*m* >0.632 and (2) check the energetic feasibility of those conformations. It is acknowledged that conformations of molecules in their pure amorphous form may not be the same as those adopted in a dispersion where the drug–polymer interactions may affect the API conformation. Based on the comparability of the *R*3*m* values determined from conformations observed in the crystal structures to those determined from CORINA predictions and MD simulations, the conformations in the amorphous API were assumed to not be dramatically different from those in amorphous dispersions with PVPVA. Future studies will investigate the influence of formulation composition on the API conformation and *R*3*m* values. Both ritonavir and lopinavir are known to be dispersible in PVPVA; an understanding of the *R*3*m* descriptor showcases that higher *R*3*m* values for molecules are representative of their interaction capability with the polymer, thus the conformations representing higher *R*3*m* values are of importance.^9^

**Figure 8:**
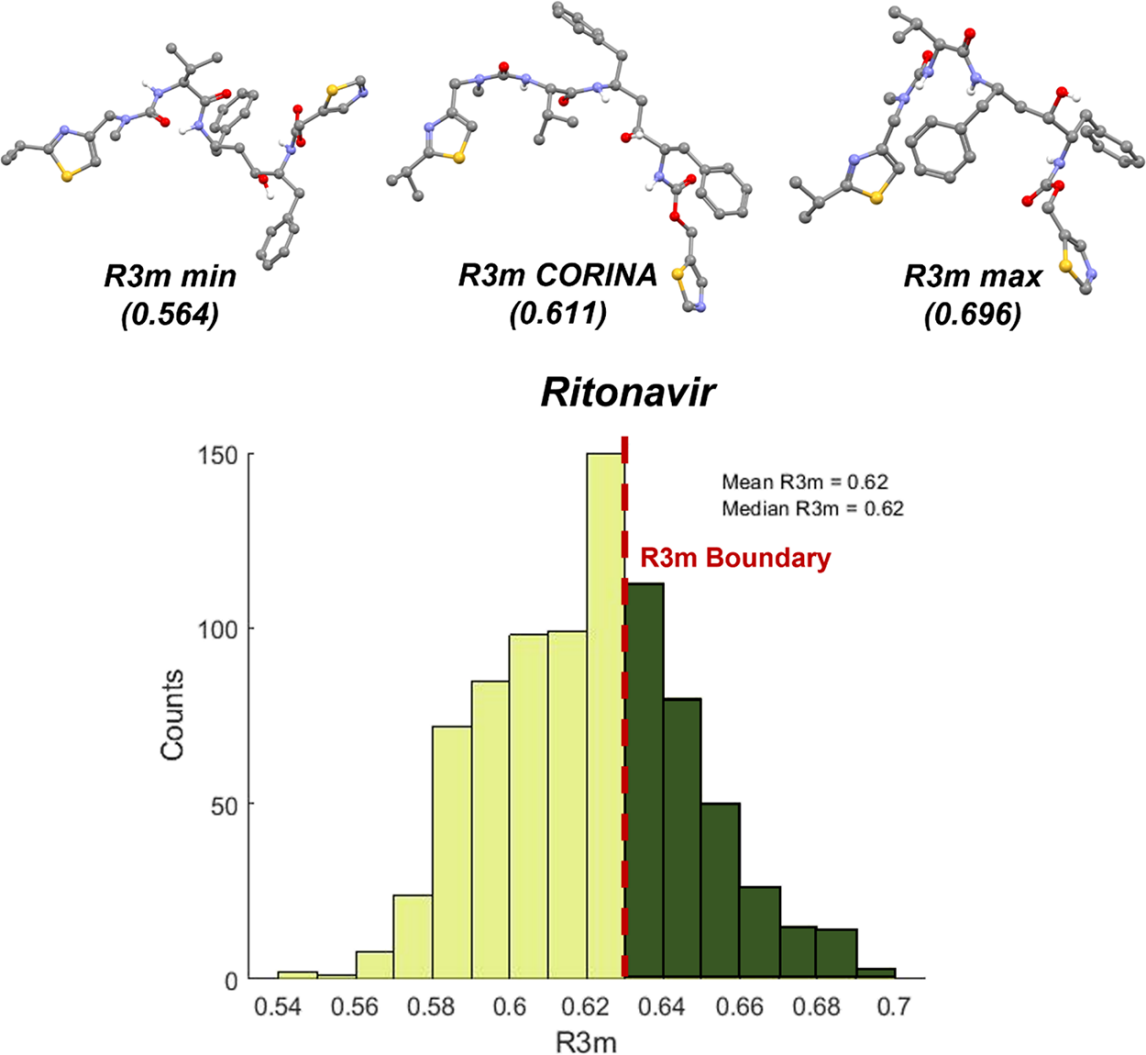
*R*3*m* distribution for 840 MD conformations of ritonavir likely in the amorphous state. The structures in conformations reflective of *R*3*m* min, *R*3*m* CORINA, and *R*3*m* max are shown (hydrogen atoms are removed for clarity except in the cases of hydrogen bond donors).

**Figure 9:**
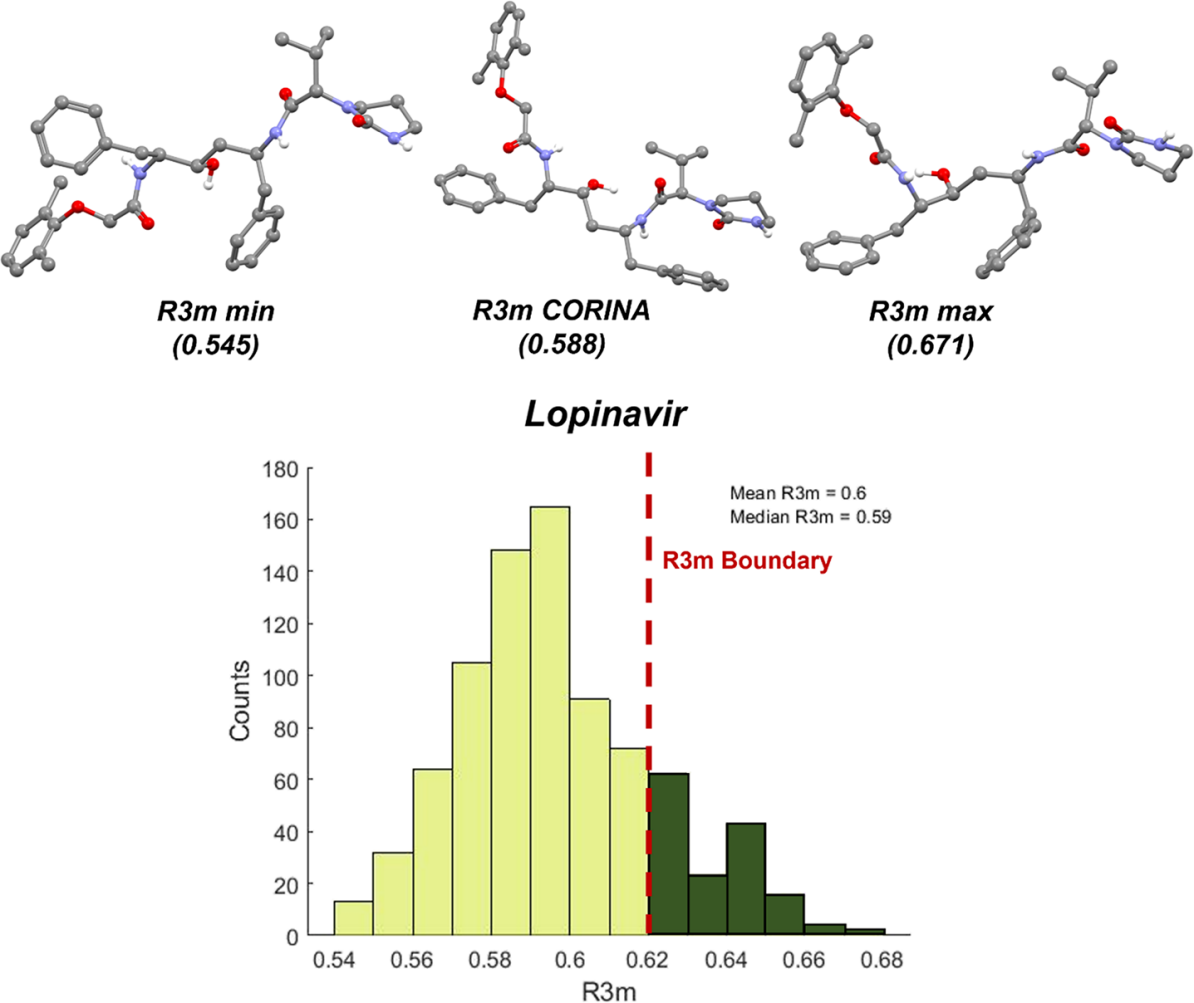
*R*3*m* distribution for 840 MD conformations of lopinavir likely in the amorphous state. The structures in conformations reflective of *R*3*m* min, *R*3*m* CORINA, and *R*3*m* max are shown (hydrogen atoms are removed for clarity, except in the cases of hydrogen bond donors).

Figures 8 and 9 show the *R*3*m* distributions calculated from MD-predicted conformations for ritonavir and lopinavir. Both distributions of *R*3*m* are broad and range from 0.54 to 0.7 (ritonavir) and 0.54 to 0.68 (lopinavir). Notably, >30% of the ritonavir conformations and >15% of the lopinavir conformations result in R3*m* >0.632. On comparison of the highest *R*3*m* (*R*3*m* max) vs CORINA (*R*3*m* CORINA) vs the lowest *R*3*m* (*R*3*m* min) conformations, it was observed that the heavier atoms were farther away and directed outward from the geometric center of the molecule for the *R*3*m* max conformation. On further investigation of individual atomic contributions to *R*3*m*, it was observed that the values for heavier atoms were different, based on the molecular conformation. For example, for the ritonavir conformation that resulted in the *R*3*m* max, the heavier N, O, and S atoms contributed 54.2% to the value of *R*3*m* whereas these same atoms contributed only 37.2% in the conformation that resulted in *R*3*m* min. Both ritonavir and lopinavir adopted conformations where heavier atoms increased *R*3*m* values above 0.632.

Consistent with previous work in this area, evaluation of the distributions of *R*3*m* for both ritonavir and lopinavir suggests that these molecules can adopt conformations that position atoms in such a way that the probability of adhesive, noncovalent interactions with the polymer PVPVA is increased.^9^ More broadly, this evaluation suggests that molecules closer to the *R*3*m* classification boundary may adopt conformations that are more or less conducive to dispersion in PVPVA. The CORINA *R*3*m* value can act as an excellent, scientifically driven starting point for predicting dispersion formation potential, but does not represent the only conformation that may be important for rationalizing the outcomes. Molecules whose *R*3*m* distributions are very close to the classification boundary enable more informed formulation decisions, by estimating the probability that they will disperse in PVPVA. This case is clear with ritonavir and lopinavir, by evaluating the threshold *R*3*m* that estimates >80% probability to successfully disperse in PVPVA. Based on Figure 7, this value occurs when *R*3*m* ≥0.654, where ritonavir and lopinavir respectively have 12.5 and 3% conformations whose *R*3*m* exceeds this value. Ultimately, it is envisioned that the *R*3*m* model could be used as a materials-sparing formulation tool that can be used to suggest the risk of proceeding with an API as a candidate for dispersion in PVPVA. Had the model based exclusively on single molecular conformations been used, both ritonavir and lopinavir would have been classified as unable to disperse in PVPVA. In contrast, the probability estimations given by the logistic regression model indicate that both API can adopt conformations that have a very good likelihood of forming a dispersion in this copolymer. In the case of a new chemical entity, this model could be used to evaluate the distribution of the *R*3*m* values for conformations in the amorphous state. If a reasonable percentage of the distribution indicated conformations that enable ASD formation, then experimental confirmation of its dispersibility in PVPVA would be warranted. Further evaluation of the accuracy of probabilities for API having median *R*3*m* values close to the classification boundary (gray region in Figure 7), should be next steps.

## 4 Conclusions

The 3D conformations that an API molecule can adopt have an impact on its *R*3*m* value because molecular shape changes influence the matrices used to calculate this molecular descriptor. Comparisons between 3D conformations obtained using the CORINA algorithm, crystal structure data, and MD simulations showed that the resulting variations in *R*3*m* were generally low for each API. Nevertheless, there are cases where changes in conformation can result in significant changes in the subsequent *R*3*m* value, as shown with aripiprazole. A large majority (86%) of an expanded data set of API had a difference in *R*3*m* ≤0.1, with a maximum difference in *R*3*m* equal to 0.28. The average absolute difference between CORINA and the median MD *R*3*m* values was 0.03, and the largest difference between the median MD *R*3*m* and CORINA *R*3*m* values was 0.11.

Ultimately, these results support the hypothesis that using MD to predict the distribution of 3D conformations library molecules can adopt results in an *R*3*m* model that better predicts dispersibility in PVPVA. In addition to greater confidence in the *R*3*m* classification boundary, by incorporating the potential for large conformational changes to impact *R*3*m*, the regression model is now able to incorporate probabilities of successful dispersion formation, which is particularly important for API having an *R*3*m* value near the model classification boundary. These findings further support the idea that *R*3*m* captures nuanced information about the API relevant to its dispersibility in PVPVA. Inclusion of conformational flexibility in future applications of *R*3*m* captures a more realistic description of the molecules and, subsequently, can allow for improved formulation decisions.

## Supporting Information Available

List of all 80 APIs used to examine the relationship between R3m calculated by CORINA-generated 3D structures and 3D structures obtained from the CCDC; a comparison of R3m values calculated from 3D conformations generated from the CORINA algorithm, obtained from crystal structure data, and generated by molecular dynamic simulations; and links to the MATLAB code for the calculation of R3m and extraction of molecule coordinates from Materials Studio .xsd files (PDF)
