# Supplementary material for: Understanding the Influence of API Conformations on Amorphous Dispersion Formation Potential Predictions using the R3m Molecular Descriptor
Source: Mol Pharm. 2024 Jan 5;21(2):770–80. doi: 10.1021/acs.molpharmaceut.3c00909 (PMC10848250; doi:10.1021/acs.molpharmaceut.3c00909)
Supplement: Supplementary file 1 — mp3c00909_si_001.pdf [file mp3c00909_si_001.pdf]

Understanding the Influence of API Conformations on Amorphous Dispersion Formation Potential Predictions using the R3m Molecular Descriptor

*Kevin DeBoyace[1,3], Mustafa Bookwala[1], Deliang Zhou[2,4], Ira S. Buckner[1], Peter L.D. Wildfong[1] **

[1]Duquesne University, School of Pharmacy and Graduate School of Pharmaceutical Sciences, 600 Forbes Ave, Pittsburgh, PA 15282

[2]Drug Product Development, Research and Development, AbbVie, 1 North Waukegan Road, North Chicago, IL 60064

[3] Current address: Pfizer Worldwide R&D, Eastern Point Road, Groton, CT 06340

[4] Current address: Small Molecules Drug Product Development, BeiGene, Beijing, China

*Corresponding author: Peter L.D. Wildfong

Address: 600 Forbes Avenue, 422C Mellon Hall, Pittsburgh, PA 15282

Telephone: 412-396-1543

Fax: 412-396-4660

Email: wildfongp@duq.edu

Table S1: List of all 80 APIs used to examine the relationship between R3m calculated by CORINA-generated 3D structures and 3D structures obtained from the CCDC. CCDC refcodes are given in parentheses.

acyclovir (MECWIC, 03)	diclofenac (MISBEW)	indinavir (DIJWOJ)	phenobarbital (PHBARB07, 08, 09, 11, 12, 13)
acetaminophen (HXACAN12)	diloxanide (DEMDIJ)	linezolid (TIYQAU01, 02)	praziquantel (TELCEU)
agomelatine (WERNOW, 01, 02)	efavirenz (AJEYAQ03)	lorazepam (BEQGIN)	pyrimethamine (MUFMAB01)
albendazole (SUTWIO)	epalrestat (ZIPKOA01, 02)	lovastatin (CPHAZO01)	raloxifene (SAQYIR)
allopurinol (ALOPUR)	eprosartan (SUJXUR)	medroxyprogesterone (MACXPR10)	rebamipide (ILUPEM)
alprazolam (MENMIB, 01)	erythromycin (QIFKEX, 01)	metaloxone (AXOGAW, 01)	risperidone (WASTEP, 01)
amitriptyline (YOVZEO)	ezetimibe (QUWYIR01)	metoclopramide (AMBZCL)	ritonavir (YIGPIO03)
aripiprazole (MELFIT01, 03, 04, 06, 07, 08, 09)	famotidine (FOGVIG06, 07)	metronidazole (MINMET02)	rofecoxib (CAXMUJ)
aspirin (ACSALA14, 20)	fenofibrate (TADLIU01, 02)	mosapride (ZEHSEK)	salbutamol (BHHPHE)
cabergoline (SUPBEK, 01, 03)	furosemide (FURSEM13, 14, 16)	mycophenolate (WAJYUC)	simvastatin (EJEQAL01, 02)
caffeine (NIWFEED03, 04)	gefitinib (FARRUM02, 03)	nabutemone (XOCUI03, 04)	spironolactone (ATPRCL01)
carvedilol (GIVJUQ, 02)	gilbenclamide (DUNXAL01)	nalidixic (NALIDX01)	sulfadiazine (SULDAZ07)
celecoxib (DIBBUL)	gliclazide (SUVGUL)	naproxen (COYRUD13)	sulfamethoxazole (SLFNMB05, 06, 07, 08)
clarithromycin (NAVSUY01, 02)	glimepiride (TOHBUN01, 02)	nevirapine (PABHIJ01)	sulpiride (PYMSBZ11)
clofazimine (DAKXUI02, 03)	glipizide (SAXFED)	niclosamide (HEBFUR01)	telmisartan (XUYHOO, 01)
clozapine (FUQMOU01)	griseofulvin (GRISFL10, 11)	nimesulide (WINWOL, 02)	theophylline (BAPLOT05, 06)
curcumin (BINMEQ08)	haloperidol (HALDOL02)	nitrofurantoin (LABJON01, 02)	trimethoprim (AMXBPM12, 13)
dapsone (DAPSUO05, 12, 15)	hydrochlorothiazide (BANPAK)	olanzapine (UNOGIN02, 03, 04)	valsartan (KIPLIG)
dexamethasone (DEXMET11)	ibuprofen (IBPRAC19)	oxcarbazepine (CANDUR01, 02)	verapamil (CURHOM)
diazepam (DIZPAM11)	imipramine (IMIPRC)	phenacetin (PYRAZB21)	warfarin (BEFZES)

MATLAB code for the calculation of R3m:
https://www.mathworks.com/matlabcentral/fileexchange/69891-r3mcalculate

MATLAB code for extracting 3-D conformation information from Materials Studio files:
https://www.mathworks.com/matlabcentral/fileexchange/70004-getatomcoords

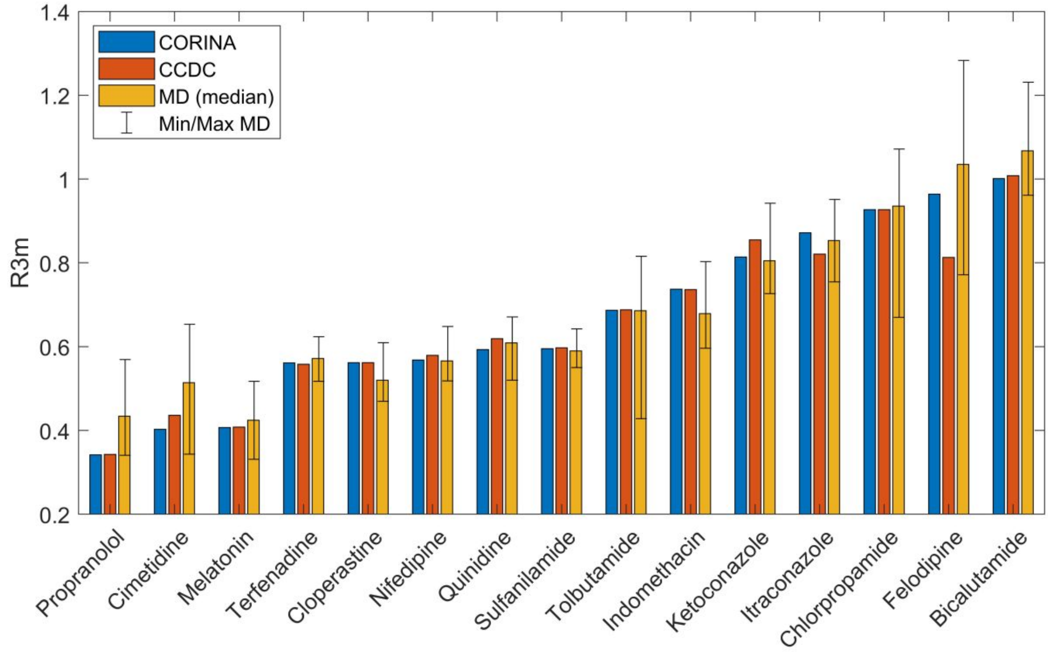

Figure S1: A comparison of R3m values calculated from 3-D conformations generated from the CORINA algorithm, obtained from crystal structure data, and generated by molecular dynamic simulations. R3m values calculated using CORINA and CCDC structures fall within the range of values determined using molecular dynamics.
